# Comorbidity in psychiatry: Way forward or a conundrum?

**DOI:** 10.4103/0019-5545.31593

**Published:** 2006

**Authors:** Nimesh G. Desai

The traditional practice of understanding and explaining all the symptoms and signs of a person presenting to mental health facilities was synchronous with the hierarchical system of classification. The clinical dictum of ‘one person one diagnosis’ was in keeping with the early classification systems wherein the diagnostic groups were arranged in hierarchy and clinicians were actively encouraged to arrive at one diagnosis for all the experiences and problem behaviours of each person. When a clinician came across a patient who had dependent pattern of alcohol use and significant depressive features, it was incumbent to decide the primary condition of the two. Research efforts were made to authenticate primary versus secondary alcoholism.^1^ In patients with alcohol or other drug dependence and an established pattern suggestive of antisocial personality profile, it was necessary to ascertain if the substance use could be explained as part of or was epiphenomenon of the antisocial personality. The underlying approach and rationale for such practice were to simplify the diagnostic groups, and to adopt one comprehensive management strategy which would be expected to benefit all symptoms. In a person with schizophrenia and depression, it was believed that effective treatment of schizophrenia would relieve the depression and, similarly, successful therapy of the antisocial personality would control the substance use, and in secondary alcoholism, the treatment of the underlying condition would take care of the treatment of alcoholism.

In 1970, Robins and Guze put forth a strong argument for making empirical evidence, through precise clinical descriptions and delineation of syndromes, the major criteria for psychiatric diagnoses.^2^ About the same time, Feinstein described the term comorbidity for all chronic diseases as ‘any additional clinical entity that has existed or that may occur during clinical course of a patient who has the index disease under study for chronic diseases’.^3^ A well-described case in point was chronic obstructive pulmonary disease in a person with diabetes mellitus. In the 1970s, the operational diagnostic criteria based on empirical, operational and measurable attributes were popularized in psychiatry initially for research^4^,^5^ and later for clinical application. The introduction of the multi-axial diagnostic criteria-based classification system with the empirical, atheoretical approach contributed to a paradigm shift. Among other contributions, the one major change was the recognition of the concept of comorbidity in psychiatry by not only permitting but encouraging the clinicians to make more than one diagnoses in a person, if the criteria were adequately met for more than one disorder. At that early stage, the DSM-III stated that one diagnosis cannot be made if it is ‘due to’ another disorder. This provision, which was gradually made more flexible, in the subsequent classification systems, and later universalized by the ICD-10 in 1992, also following the same approach, modified not only the coding and record keeping, but more significantly broadened the perspectives of clinicians. The assessment of mental health problems of persons with multiple diagnoses, and the related implications in planning treatment and management strategies, has been significantly influenced during the 1980s and 1990s by this one paradigm shift. Soon after DSM-III, in path-breaking research, Boyd *et al.* in 1984, reported from a large community-based sample in USA that there was ‘a general tendency toward co-occurrence, so that the presence of any disorder increased the odds of having almost any other disorder’.^6^ They defined comorbidity in epidemiological terms as ‘the relative risk of a person with one disorder, to receive the diagnosis of another disorder’. The current systems of DSM-IV and ICD-10 actively encourage multiple diagnoses in the same person, regardless of the possible contribution to aetiology, allowing the maximum amount of diagnostic information. In 1990, Burke defined comorbidity as ‘the presence of more than one specific disorder in a person in a specified period of time’.^7^

## COMORBIDITY: THE DEBATE

It is not as if the phenomenon of comorbidity is entirely new, or that suddenly human beings have begun to experience and manifest multiplicity of problems! Human beings have always experienced and presented with overlapping problems of psychological and/or behavioural nature. It is the scientists and professional groups who have changed the fundamental approach to understand these problems. In the new scenario, professional perspectives and definitions have changed. It has been persuasively argued by Maj that psychiatric comorbidity is a byproduct of the recent diagnostic systems.^8^ The four determining factors he has outlined are: (i) the rule laid down in the construction of DSM-III that the same symptom could not appear in more than one disorder; (ii) proliferation of diagnostic categories; (iii) limited number of exclusionary hierarchical rules; and (iv) the application of operational diagnostic criteria.^8^ The issue of real versus apparent comorbidity had been raised by Feinstein early enough, and it is well established that in research studies, hospital samples generate higher rates of comorbidity as compared to community samples.^9^ On the other hand, it is also well established that clinicians, in their practice, do not adequately capture the complexity of comorbidity. Pincus et *al.* have described the potential usefulness of involving multiple diagnoses (comorbidity), but also argued that ’while it makes intuitive sense that more comprehensive diagnostic information would result in improved patient outcomes, we do not have studies that have investigated this issue’.^10^ It is likely that psychiatrists in clinical practice are simply choosing not to make certain diagnoses because they do not consider them to be a clinically relevant focus of treatment.^11^ This seems particularly relevant for making diagnoses of personality disorders on Axis II, understandable in the context of relatively lesser evidence for the effectiveness of treatment for personality disorders, and the availability of such treatment methods. This is considerably important for developing countries where the human resource situation, and lack of appropriate data-capturing health information systems, may render the growing complexity of comorbidity impracticable, if not irrelevant.

The broad range of comorbidities seen across Axes I and II disorders as per the current classification systems is in itself quite diverse for any unitary explanation or hypothesis for psychiatric comorbidity, notwithstanding the medical comorbidity with psychiatric disorders. The multifarious complexity has been examined in various interpretations, viz. that individuals actually suffer from multiple disorders, all disorders are offspring of a defective persona, there is one common biological mechanism which leads to more than one disorder, and that disorders are reactions of the individual vulnerabilities to noxious stimuli.^14^ There have been other explanations of the complexity of psychiatric comorbidity.^8^,^15^

In simpler terms, the four explanatory models or causative mechanisms that are described here can be sufficient to understand a variety of comorbidities. One, disorder A predisposes the individual to disorder B, e.g. anxiety disorders predisposing a person to alcohol or other drug use disorders. Two, either of the two disorders A or B may predispose the individual to the other disorder, e.g. anxiety and depression, alcohol and depression. Third, one underlying biological mechanism may contribute to two or more disorders, e.g. serotonergic mechanism for depression and obsessive–compulsive disorder. Fourth, Axis II personality disorder or mental subnormality may predispose an individual to Axis I disorders. The conceptual and deterministic issues of comorbidity continue, and are likely to continue, to be debated but the predominant emerging view is that different sets of comorbidities require to be understood separately.^16^ Herein, the possible future direction is also indicated, by which the valid and enduring comorbidity categories will be retained and the others will be reviewed and dealt with by being discarded or grouped with other categories.^11^,^17^,^18^

The current practice of obtaining the maximum yield from clinical and research settings for comorbidity rates and patterns has led to astounding figures of overall psychiatric morbidity. The Epidemiological Catchment Area (ECA) Study reported in 1990, lifetime prevalence rates of 22.5% for any non-substance abuse mental disorder, 13.5% for alcohol dependence abuse, and 6.1% for other drug dependence abuse, totalling over 40% lifetime prevalence rates for all disorders put together for the American population. In the National Comorbidity Survey (NCS) reported in 1994, nearly 50% of the respondents in a national probability sample of the USA^20^ had one or more diagnosable mental disorder or substance use disorder, a finding replicated and reported in 2005.^21^ Methodological and public policy issues have been raised for these trends,^22^ and more importantly it has been opined that ‘the current strategy of diagnosing “maximal” comorbidity may not result in “optimal” comorbidity in terms of best clinical practice. The practice of listing multiple diagnoses has the power to both enhance and obfuscate important clinical information.’^22^ It may well be said that the current predominance of ‘splitters’ is expected to give away to ‘lumpers’, paving the way for a more appropriately balanced position.^23^ The tradition of splitting and maximizing the output seems to be largely DSM-based, and from the American Research, although similar trends have been reported from the UK and Australia too.^24^–^27^ The American trend of referring to Axis I psychiatric disorder with substance use disorder as ‘dual diagnosis’ seems to be less favoured, to the tendency of referring to all these as comorbidity. The clinical usefulness and implications of ‘triple diagnosis’ for persons with psychiatric disorder, substance use disorder and HIV-positive status/clinical AIDS, is already well appreciated. It may be argued that, in addition to the conceptual and scientific reasons, the high rates of dual diagnoses and comorbidity are indicators of the American way of life and social fabric but it cannot be easily denied that global trends would be the same sooner or later.

## COMORBIDITY: THE CHURNING CONTINUES

The tremendous implications of the emerging findings of comorbidity across all groups of psychiatric disorders for the clinical issues of diagnostic practices, variability in treatment response, and unpredictability of the course are likely to shake the very foundations of clinical psychiatry. The implications for research not only of clinical nature, but also of genetic nature are profound.^28^

It can be easily seen that the research till date has only just begun to address some of the many issues of the subject, and many of the issues remain unexplored, as well as unanswered. The fundamental nature of stirring up and shaking up of the concepts and practices in psychiatry, which are emerging on the horizon do not suggest a smooth road ahead. It may be good to recognize that although comorbidity has opened up new challenges, the way forward is not a clear path. Indeed, the current research suggests that the field is on the brink of a conundrum with unpredictable results, not unlike the mythological Indian descriptions of *samudra-manthan,* i.e. churning of the ocean, expected to yield nectar and/or poison! It will have to be seen if these developments and the churning in the conundrum will benefit science and more importantly the end users—the persons with mental health problems and their family members. It can safely be said as the churning continues—that the last word has not been said, indeed far from it!
